# Microbe Profile: *Ehrlichia ruminantium* – stealthy as it goes

**DOI:** 10.1099/mic.0.001415

**Published:** 2023-11-23

**Authors:** Damien F. Meyer, Amal Moumène, Valérie Rodrigues

**Affiliations:** ^1^​ CIRAD, UMR ASTRE, Centre for Research and surveillance on Vector-borne diseases in the Caribbean, WOAH Reference Laboratory for Heartwater, F- 97170 Petit-Bourg, Guadeloupe, France; ^2^​ ASTRE, CIRAD, INRAE, Univ Montpellier, Montpellier, France

**Keywords:** effectors, Ehrlichia, intracellular bacteria, outer membrane protein, pathogenesis, type IV secretion system

## Abstract

*
Ehrlichia ruminantium* is an obligate intracellular pathogenic bacterium that causes heartwater, a fatal disease of ruminants in tropical areas. Some human cases have also been reported. This globally important pathogen is primarily transmitted by ticks of the *Amblyomma* genus and threatens American mainland. *E. ruminantium* replicates within eukaryotic mammal or tick cell is a membrane-bound vacuole, where it undergoes a biphasic developmental growth cycle and differentiates from noninfectious replicative form into infectious elementary bodies. The ability of *E. ruminantium* to hijack host cellular processes and avoid innate immunity is a fundamental, but not yet fully understood, virulence trait of this stealth pathogen in the genomic era.

## Graphical abstract

Summary of *Ehrlichia ruminantium* infection. *E. ruminantium* bacterium is typically spread to ruminants by bite from infected ticks. Following adhesion of infectious elementary bodies to host cell, *E. ruminantium* establishes the depicted intracellular biphasic lifestyle by the means of secreted effector proteins that hijack host cellular pathways. Replicating bacteria (reticulate bodies) fill a large cytoplasmic vacuole named morula inside mammalian or tick cells. Formation of this vacuole is critical for bacterial development and subsequent lysis of the host cell leading to the establishment of heartwater disease. Genetic diversity is one remarkable and biologically significant feature of pathogens in the *Anaplasmataceae* family, including *E. ruminantium *. The maximum-likelihood phylogenetic tree of four representative species of *Ehrlichia* (* E. chaffeensis* str. Arkansas, *E. canis* str. Jake, *E. muris* AS145, *E. ruminantium* str. Gardel) with *Wolbachia* endosymbiont of D. melanogaster as an outgroup was reconstructed on the basis of concatenated nucleic acid alignment of proteins shared by all species (core genomes) with 100 bootstrap resamplings. Major hosts of these bacteria are indicated. Mb, megabase. ORFs, open reading frames. (All images created by S. Mateus, C. Noroy. O. Gros and D.F. Meyer.) 
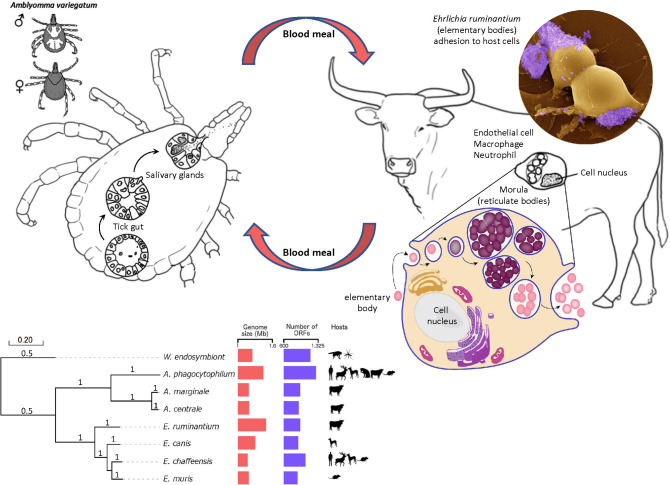

## Taxonomy

Domain Bacteria, phylum Proteobacteria, class Alphaproteobacteria, order *Rickettsiales *, family *Anaplasmataceae *, genus *Ehrlichia *, species *ruminantium* [1, 2].

## Properties

*Ehrlichia ruminantium* is a Gram-negative polymorphic bacterium (ranging from 0.2 to 2.0 µm in diameter) and exhibits a bi-phasic developmental cycle. The infectious extracellular elementary body (EB) enters host cells via pathogen-orchestrated receptor-mediated uptake to reside within a host cell-derived vacuole. EB transitions to the replicative reticulate body (RB) that divides by binary fission. RB redifferentiate to EB that subsequently exits to reinitiate the infection cycle. *E. ruminantium* replicates within two hosts, ruminants and vector ticks, by orchestrating refined adaptative molecular strategies. Upon sensing environmental cues, *E. ruminantium* secretes effector proteins into the host cell to evade innate immunity and control host-cell metabolism.

## Genome

The genome of several strains of *E. ruminantium* have been sequenced since the first ones in 2005 and 2006 [1, 2], allowing to decipher the molecular bases for pathogenesis and virulence attenuation. These sequences revealed small contracted genomes with an average size of about 1.3 Mb and coding up to about 1200 proteins. All *E. ruminantium* isolates have a chromosome that display the lowest coding ratio (~64 %) observed among bacteria due to long intergenic regions. These genomes show an active process of expansion/contraction targeted at tandem repeats in noncoding regions. No plasmids are present in *E. ruminantium* Gardel, Welgevonden and Senegal strains. Genomic comparisons between virulent and attenuated strains of Senegal isolate revealed that *ntrX* gene is a probable virulence attenuator gene [3]. This finding provides a genetic basis for the generation of attenuated strains for vaccine production, with *ntrX* serving as a promising target for mutagenesis approaches [3]. A theoretical model based on game theory elucidated the host-triggered evolutionary process leading to the attenuation of obligate intracellular bacteria [4]. High-throughput sequencing technologies allow now cost-effective whole-genome sequencing of numerous *E. ruminantium* strains isolated in cell culture for phylogeny and phylogeography studies.

## Phylogeny

*Ehrlichia ruminantium* is one of the nine species of *Ehrlichia *, and is most closely related to *Ehrlichia canis *, an intracellular pathogen of dogs and humans. Other closely related *Anaplasmataceae *, also pathogenic on humans, are *Ehrlichia chaffeensis* and *Anaplasma phagocytophilum. E. ruminantium* constitutes a single species but numerous field strains have been isolated from infected ruminants or ticks. Recombination between *E. ruminantium* strains is proposed to be a major driver of genetic diversity in this obligate intracellular pathogen [5]. This genomic variation inside *E. ruminantium* species could explain the limited efficacy observed with vaccinal strains in the field, although precise correlations are lacking.

## Key features and discoveries

The first mention of heartwater in the literature was made in 1838 by Louis Trichardt after a trek in South Africa where he lost several sheep 3 weeks following a severe tick infestation. Eighty-seven years later, the Canadian bacteriologist Edmund Cowdry identified the bacterial pathogen causing the disease and named it *Rickettsia ruminantium*. That name was changed later to *Cowdria ruminantium* and more recently to *Ehrlichia ruminantium* thanks to molecular phylogenetics. The disease is responsible for a significant economic burden, particularly in regions where it is endemic, such as Sub-Saharan Africa and some Caribbean islands. Heartwater has also been reported on islands in the Indian Ocean and the Atlantic Ocean. The estimates total animal production losses from the disease to the Southern African Development Community only are thought to average US$48 million annually considering acaricide use, production losses, antibiotic treatments, with up to 90 % mortality rate in naïve animals (source https://www.woah.org/en/disease/heartwater/). The predominant clinical signs of heartwater, resulting from severe damage to the vascular endothelium, include lung and brain congestion, hydrothorax and hydropericardium. In the early stages of the disease, animals can be treated with sulfonamides and tetracyclines. Additionally, experimental vaccines (recombinant, attenuated and inactivated) have been developed, showing promise. However, their effectiveness has been limited due to the genetic and antigenic diversity of *E. ruminantium* strains in the field. Controlling heartwater requires robust vector control measures, including restricting animal movement, tick population control, identification and elimination of infected animals.

*E. ruminantium* is maintained in nature in a zoonotic cycle between ticks, primarily *Amblyomma variegatum* and *Amblyomma hebraeum* even if other *Amblyomma* spp. can also transmit *E. ruminantium *, and persistently infected hosts. However, some recent data show that in West Africa, *Rhipicephalus microplus* also acquires and transmits *E. ruminantium *, causing mild disease in sheep compared to the natural vector *A. variegatum* [6].

The understanding of the molecular pathogenesis of *E. ruminantium* has been significantly advanced through a series of recent key discoveries.

The thorough identification and characterization of outer membrane proteins (OMPs) have unveiled their critical role in the bacterium’s virulence and pathogenesis, offering valuable targets for the development of effective vaccines. By developing an approach to extract and analyse OMPs from infectious EBs, a study identified 18 unique OMPs, encompassing proteins involved in cell structure, biogenesis, transport/virulence, porins, and proteins with unknown functions. This first comprehensive proteome characterization of the outer-membrane fraction in *Ehrlichia* spp. represented a pioneering effort, providing novel insights into the organism’s pathogenesis [7].

Furthermore, the investigation of the OMP Ape (adhesion protein of *Ehrlichia *) has shed light on its involvement in the early adhesion process to host bovine aortic endothelial cells (BAEC). The recombinant form of the protein demonstrated adherence to BAEC-coated beads, suggesting its potential role in the initial interaction between *E. ruminantium* and host cells. Additionally, the interaction of Ape with proteins from various cellular fractions further supports its relevance in host-cell adhesion. The observed reduction in bacterial numbers after enzymatic treatment targeting surface glycosaminoglycans on BAEC highlights the involvement of these molecules in *E. ruminantium* adhesion. However, the full complement of adhesin-receptor pairs and how they mechanistically drive ehrlichial cellular entry into endothelial cells are yet incompletely defined [8].

The defining feature of *E. ruminantium *’s intravacuolar lifestyle is the secretion of effector proteins into host cells to establish an acidic (pH ~5.0) replicative niche. These effectors interact with eukaryotic proteins and genes to manipulate host cellular signalling pathways, enabling the bacteria to evade degradation and acquire the necessary nutrients for safe intracellular replication. Erip1 (* E. ruminantium* injected protein 1), a new substrate of the type IV secretion system (T4SS) was recently discovered in *E. ruminantium* using SATE 2.0 prediction software (www.sate.cirad.fr). This novel bacterial effector is crucial for *Ehrlichia* infection and undergoes tyrosine phosphorylation and nuclear import to target the host cell’s innate immune response.

The regulatory role of ErxR (* E. ruminantium* expression regulator) has been elucidated in response to iron availability. Exposure of *E. ruminantium* to iron starvation induced the expression of ErxR, subsequently leading to the upregulation of genes associated with the T4SS and outer membrane proteins of the Map family. This tight co-regulation provides insights into how *Ehrlichia* senses changes in iron concentrations in its environment and adjusts the expression of virulence factors accordingly, advancing our understanding of the bacterium’s infection process [9].

Lastly, the prediction and analysis of type IV effectors (T4Es) in *Ehrlichia* spp. have unveiled a conserved core effectome shared among all strains. Strain-specific candidate T4Es were identified, predominantly located in gene-sparse regions of the genomes and showing non-canonical GC% content. This repertoire of predicted effectors serves as a foundation for future functional characterization, facilitating the investigation of their precise roles in *Ehrlichia* spp. virulence and host adaptation [10].

These findings greatly enhance our comprehension of *E. ruminantium* pathogenesis, offering valuable insights for the development of effective vaccines and host-targeted antimicrobial approaches to combat this stealth intracellular bacterium and mitigate its impact.

## Open questions

- What host and environmental signals trigger *E. ruminantium* to adhere to and invade host cells, activate its T4SS, secrete bacterial effectors, and ultimately enable it to hijack innate immunity, withstand lysosomal defenses, and replicate within the vacuole?
- Is quorum sensing involved in *E. ruminantium* infection, notably in the regulation of EB to RB synchronous conversion during intracellular growth, as well as EB redifferentiation and the triggers for host-cell lysis?
- How do *E. ruminantium* strains interact with the tick immune system and other microbial species, particularly other *Anaplasmataceae *, inside tick cells?
- What are the major drivers of *E. ruminantium *’s virulence and field genotypic heterogeneity, and how *E. ruminantium* diversity is affected by geography, vector availability and host communities?
- What are the underlying mechanisms governing the genomic plasticity and host specificity of *E. ruminantium *?
